# Protective effect of blackthorn fruits (*Prunus spinosa*) against tartrazine toxicity development in albino Wistar rats

**DOI:** 10.1186/s13065-019-0610-y

**Published:** 2019-08-09

**Authors:** Igori Balta, Bogdan Sevastre, Vioara Mireşan, Marian Taulescu, Camelia Raducu, Adina Lia Longodor, Zamfir Marchiş, Codruta Stefania Mariş, Aurelia Coroian

**Affiliations:** 1 Department of Toxicology, Faculty of Animal Science and Biotechnology, University of Agricultural Sciences and Veterinary Medicine, Calea Manastur 3-5, 400372 Cluj-Napoca, Cluj Romania; 2 Department of Physiopathology, Biology, Breading and Pathology of Laboratory Animals, University of Agricultural Sciences and Veterinary Medicine, Calea Manastur 3-5, 400372 Cluj-Napoca, Cluj Romania; 3 Department of Anatomy and Physiology, University of Agricultural Sciences and Veterinary Medicine, Calea Manastur 3-5, 400372 Cluj-Napoca, Cluj Romania; 4 Department of Pathology, Necropsy Diagnosis, Forensics, Oncology, University of Agricultural Sciences and Veterinary Medicine, Calea Manastur 3-5, 400372 Cluj-Napoca, Cluj Romania; 5 Department of Food Technology, University of Agricultural Sciences and Veterinary Medicine, Calea Manastur 3-5, 400372 Cluj-Napoca, Cluj Romania; 6 Department of Horse Breeding Technology, University of Agricultural Sciences and Veterinary Medicine, Calea Mănăștur 3-5, 400372 Cluj-Napoca, Cluj Romania; 7 Environment and Soil Science Department, University of Lleida, Av. Alcalde Rovira Roure 191, 25198 Lleida, Spain

**Keywords:** Tartrazine toxicity, Blood biochemistry, Hematology, Rat histopathology, *Prunus spinosa*

## Abstract

**Background:**

Tartrazine (Yellow 5 or E102) is a synthetic food dye able to modify perception and behavior, causing agitation, confusion, rhinitis and can produce hyperactivity syndrome in children when is combined with benzoates. Additionally, it can trigger oxidative stress which consequently generates metabolic disorders. Therefore, the study was designed to evaluate the harmful effects of the food additive tartrazine and to observe beneficial properties of blackthorn fruits (*Prunus spinosa*) on the blood and organs of albino Wistar rats.

**Materials and methods:**

This study was carried out on 20 mature Wistar rats, randomly divided into four groups of five animals. Over the course of the experiment, the control group received only food and drinking water, group I received 75 mg of tartrazine dissolved in (250 ml) water group II was given 75 mg of tartrazine and 200 mg of dried blackthorn fruit powder 200 mg dissolved simultaneously in (250 ml) of tartrazine-water mixture (aiming to reduce the tartrazine toxicity) and group III received a higher dose of tartrazine (100 mg) in (250 ml) of water.

**Results:**

At the end of the experiment, values regarding kidney and liver weight were significantly increased, while the weight of the spleen was slightly decreased compared with the weight of the control group. Biochemical and hematological assays, of the blood samples show that the addition of tartrazine in the diet of rats caused significant changes in all biochemical and hematological parameters of the blood. In the group II, which received (*P. spinosa*) powder combined with tartrazine, the biochemical and hematological parameters had average values similar to the control group.

**Conclusions:**

Histopathological assay showed that the application of tartrazine in the group I, II and III produced lesions of the kidneys, spleen and the liver for all rodents. Tartrazine was able to generate histopathological changes, which caused significantly tissue lesions of the liver and significant changes in blood parameters. Blackthorn powder showed a promising protective role for the blood parameters but demonstrated no significant benefits for the organs.

## Introduction

The meaning of food additive can be defined in a simple way—it is a substance that is not a normal constituent of food, and is added intentionally for a technological, organoleptic or nutritional purpose [1]. As a class of the food additives category, synthetic food dyes should be subjected to numerous investigations such as biochemical, mutagenic and acute toxicity experimental studies of both long and short duration for maximal consumer safety [2]. Using food dyes in the food industry today can produce unintended consequences in terms of safety. Some studies demonstrated the allergenic [3–6], clastogenic [7–9] mutagenic and carcinogenic effects on different dyes, such as: tartrazine, amaranth, Sudan IV, carmoisine, brilliant blue [8]. Tartrazine is a synthetic lemon yellow azo dye primarily used as a food coloring. It is obtained by chemical synthesis from coal tar, and is widely used in the food, cosmetic and pharmaceutical industry [10]. This food additive is linked with provoking asthma attacks and hives in children [6, 11]. It can also modify perception and behavior, causing agitation, infertility, confusion, rhinitis, migraine [12]. Furthermore, in children, when it is combined with benzoates it could produce hyperactivity syndrome [13] and thyroid tumours [14]. Doses of 0.14 up to 750 mg of tartrazine in people produce effects on peripheral nerves, paresthesia and teeth change. After consuming tartrazine people suffering from asthma, may have side effects, because the tartrazine triggers mast cell degranulation [15–19]. This leads to the release into the body of substances stored in granules (histamine) within the mast cells, which in turn causes the symptoms of an allergic attack.

FAO/WHO established ADI (an acceptable daily intake) for tartrazine of 0–7.5 mg/kg b.w./day [20]. Tartrazine exposure by inhalation is considered hazardous. This dye is responsible for inducing oxidative stress in rats [21] and that, is associated with free radical production, decreasing significantly biochemical serum parameters and antioxidants of blood. Onion juice (*Allium cepa*) added in the rat diet reduces the toxic effect of tartrazine on the enzymatic activity of antioxidants and biochemical serum parameters. The lethal dose in Wistar rats is >2000 mg/kg/b.w. reported by EFSA and others [20, 22]. Treating pregnant females of mice with 68 mg/kg-b.w. of tartrazine after evaluation of the females together with embryos, presented cytogenetic changes and females had increase in chromosomal aberrations [23]. The dye is able to increase levels of hyperactivity and aggression in the behavior of hyperactive children [24] and induce damage in the thyroid glands of rodents [25, 26]. In mammalian cells, tartrazine can trigger chromosomal aberrations in bone marrow and can cause an increase of sister chromatid exchanges (SCEs) [9]. Ordinarily, 4–5 sister chromatid exchanges occur for a chromosome pair over one mitosis, whilst 14–100 (SCEs) is not a normal variable and can present a hazard to the organism. A recent study, [27] demonstrated that this synthetic food additive increases the toxicity rate, genotoxicity, mutations and disruption of sex ratio in Drosophila melanogaster model. However, other studies have shown that tartrazine has a potential genotoxic [28] and clastogenic activity.

In Wistar rats and mice the tartrazine can induce chromosomal aberrations in bone marrow [9]. Additionally, other studies have shown that tartrazine has a genotoxic potential effect on leucocytes of rats [28], produces damage in bone marrow cells and affects the DNA in the liver and kidneys of rats [29]. Cytotoxic activity was observed at concentrations of 1, 2, 4 and 8 mM on human peripheral blood cells [30].

In the study realized by Mehedi et al. [31] was demonstrated that sub-chronic ingestion of tartrazine at low doses added in water could cause a structural alteration of the spleen and intestine, inducing a depressing effect on the humoral immune response in male mice. According to recent findings [28, 32] tartrazine is able to generate ROS (reactive oxygen species), accelerating oxidative stress, modifying the biochemical profiles and structure in the renal and hepatic tissues. After histology examination of male albino rats [32], their livers showed diffuse vacuolar degeneration in the hepatic parenchyma, showed hepatic congestion and hyperplasia in the bile duct epithelium; and in the hepatocytes of some individuals, necrobiosis was detected. The kidneys showed a mononuclear leukocyte infiltration in the renal cortex, a perivascular edema with infiltration of inflammatory cells, congestion of the renal blood vessel with necrobiotic changes.

Blackthorn or sloe (*Prunus spinosa*) is a spiny shrub from Rosaceae family, is native to Europe, western Asia and northwest Africa, which was locally naturalized in North America and New Zealand [33]. This plant prefers growing on rocky hills, cliffs, forest edges, pastures and it can be found from the plains to the mountain floor (1000–1600 m) [34]. The flowers are rich in organic acid, flavones, quercetin, kaempferol, magnesium, potassium and glycosides. The fruits of this tree has a high content of polyphenols, sugars, vitamin C, calcium and magnesium salts, organic acids, beta-sitosterol, ferulic acid, anthocyanins, prunicyanine, gumiresines and tannins [35].

It is known that the fruit exerts astringent, diuretic, anti-diarrheal and antidysentery effects. They are recommended for stomach pain, diarrhea, kidney disease, dysentery, biliary dyskinesia, convulsive cough, diseases of cardiovascular system and for digestion stimulation [36, 37]. Its phytotherapeutic properties can be attributed to various bioactive molecules such as polyphenolic and anthocyanin compounds [35, 36]. Ethanol extracts from sloes of (*Prunus spinosa*) have a high content of phenolic substances and a high antioxidant activity and it can be used in pharmaceutical and food industry [38].

Polyphenols with anthocyanins have a powerful antioxidant activity, behaving as free radicals in the body, reducing significantly harmful effects of free radicals which are produced by reactive oxygen species [39, 40]. Based on these findings and the fact that *Prunus spinosa*/blackthorn fruits are a reach rich source of antioxidants, and may exert a potent protective effect in Wistar albino rats, fine fruit powder was included in this study to investigate the curative effects of blackthorn against the impact produced by tartrazine [41–43]. Therefore, the aims of this study were to evaluate the toxicity of the food additive tartrazine and the protective effects of (*Prunus spinosa*) powder on hematological, biochemical parameters of blood and damage that was induced in organs of Wistar albino rats, after supplementation of tartrazine (dissolved in water) daily over the course of 7 weeks.

## Methods

The tartrazine study was conducted over a 7-week period, in which albino Wistar rats received food and substances daily ad libitum. Before the experiment started, rats were acclimatised for 2 weeks under the following conditions: (12/12 light–dark cycle, humidity 50 ± 10%, temperature 22 ± 1 °C), in the Establishment for Laboratory Animals of the University of Agricultural Science and Veterinary Medicine, Cluj, Romania. They were allowed to free access to standard food and water. All the experimental procedures comply with Directive 2010/63/EU and national legislation (Law no. 43/2014) [44, 45]. The project was approved by the institutional Research Ethics Committee (approval no. 110/23.04.2018) and authorized by regional state veterinary authority (authorization no. 8187/18.04.2018).

### Experimental design

Animals were randomly divided into 4 groups and each group had 5 animals per cage. The treatment were: (I) control group (C) received only food and drinking water for the full term of experiment; (II) group I received 75 mg of tartrazine dissolved in 250 ml of water; (III) Group II received 75 mg of tartrazine dissolved in drinking water and further, 200 mg of (*Prunus spinosa*) fine powder was added and mixed thoroughly in the tartrazine–water mixture (250 ml); (IV) group III was subjected to the highest concentration of tartrazine (100 mg) in water (250 ml). The dry extract of (*Prunus spinosa*) was administrated only in the diet (drinking water) of group II in order to diminish toxic effects of tartrazine. After 7 weeks of administering tartrazine, the individuals were again weighed.

### Chemicals

**Fig. 1:**
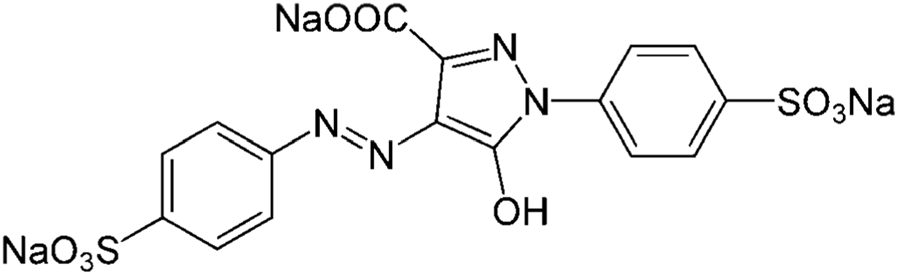
Chemical structure of tartrazine (E102) [52]

Tartrazine (Fig. 1), Trisodium-5-hydroxy-1-(4-sulfonatophenyl)-4-(4-sulfonatophenylazo)-Hpyrazole-3-carboxylate, an azo dye as orange colored powder (CI Number = 19,140, EEC Number = E-102), with molecular formula C_16_H_9_N_4_Na_3_O_9_S_2_ is water-soluble and meets the standardized requirements. From the present study, tartrazine was purchased from Stera Chemicals (Bucharest, Romania) [46, 47].

### Fruit samples

Fresh blackthorn fruits were collected at full maturity at the end of September, from the (45°08′23.8″N 24°15′23.8″E) Vlăduceni, Commune Păușești Măglași, Vˆalcea County, Romania. *Prunus spinosa* fruits were recognized by horticulturist Dr. Adrian Vescan, at the Department of Horticulture on the territory of the University of Agricultural Sciences and Veterinary Medicine, Cluj-Napoca. The *Prunus spinosa* fruits were chosen due to their beneficial properties of bioactive compounds [41–43]. Plant material was washed, the stones (seeds) were removed, and proper pulp was dried at 30 °C in a drying oven (Memmert Lab Oven UFB 400 1400 W, Germany). After that, obtained dried pulp was milled in order to achieve the proper pulp fine powder.

### Blood sample and organ collection

Finally, all animals were subject to deep narcosis using isoflurane and blood samples were collected from the orbital sinus for biochemical and hematology analysis. Blood samples were collected in vacutainers containing EDTA. Later the animals were euthanized by prolonged narcosis, the animals were considered dead when no heart and respiratory activity was recorded. The irreversibility of phenomena was assured by cervical dislocation. Organs such as liver, spleen, kidneys and brain were collected and frozen at a − 20 °C in order to realize histopathological examinations.

### Clinical biochemistry

Biochemical determinations of blood samples were performed with a semiautomated screen-type biochemical analyzer STAT-FAX 1904 Plus, Global Medical Instrumentation, Inc., 6511 Bunker Lake Blvd., Ramsey Minnesota, 55303 USA. The following parameters were measured: Aspartate aminotransferase (AST), Alanine aminotransferase (ALT), Glucose (GLU), Cholesterol (COL), Triglyceride (TG) and Creatinine (CRE) using special kits following the producer specifications [48].

### Hematology

Hematological measurements were performed by using 5-Part Differential, performed on the Abacus Junior Vet Analyzer, Diatron, (Messtechnik, Budapest, Hungary). This device evaluated 18 hematological parameters, from (*n *= 20) samples, using (*n *= 5) replicates for each group requiring just 25 µl of blood sample.

### Histopathology

Samples from liver, kidney, spleen and brain were collected and fixed in 10% phosphate-buffered formalin for 24 h, routinely processed, embedded in paraffin wax, cut into 3–4 µm sections and stained with hematoxylin and eosin (H&E). Samples were examined using an Olympus BX51 microscope. Photomicrographs were taken using Olympus SP 350 digital camera and CellˆB basic imaging software (Olympus Corporation, Japan) [29]. Histological changes, including degenerative processes, necrosis, inflammation, and cell proliferation were evaluated in all samples.

### Statistical analysis

The normal distribution of the values for each biochemical parameter (AST, ALT, glucose, cholesterol, triglyceride, creatinine), hematological parameters (WBC, LYM, MON, NEU, LYM, MO, NE, RBC, HCB, HCT, MCV, MCH, MCHC, RDWc, PLT, PCT, MPV, PDWc) and morphological parameters (initial and final weight, liver, right and left kidney, spline and brain weight) were verified using the Shapiro–Wilk test. The average for each parameter is the arithmetic mean of five replications and it was examined using data from five repetitions with an ANOVA model (JMP version 12, SAS Institute, USA) that included terms for biochemical, hematological and histopathological parameters. Significant differences among groups (control, I, II and III) means were further examined using Tukey’s multiple range test at the 0.05 probability level. A *p*-value of 0.05 was used as the threshold for statistical significance.

### Determination of total phenolics

The total amount of polyphenols in Blackthorn fruit extracts was investigated using adjusted Folin–Ciocalteu colorimetric method [49, 50]. The method is based on the polyphenol reducing properties of the hexavalent molybdenum of the polyphosphomolybdate contained in the Folin–Ciocalteu reagent. Hexavalent molybdenum is partially reduced by polyphenols in a strongly acidic medium at a lower valence state (+ 4, + 5), which in alkaline medium is colored in blue (presenting absorption bands at 750 nm). Absorption is formed due to the charge transfer bands, specific for transition metals situated in low valence states in the mixture. Folin–Ciocalteu reagent (2 M) is diluted 1:10 and in 7.5% sodium carbonate solution for neutralization and alkalinization of the reaction medium. Preparation of the calibration curve was achieved by mixing 2.5 ml of Folin–Ciocalteu reagent diluted 1:10 or with 0.2: 0.4; 0.6; 0.8; 1.2 µM/ml gallic acid. The initial yellow solution Folin–Ciocalteu slightly becomes green once with the increase in gallic acid concentration. After aprox 10 min, the time required to complete the redox reaction, 2 ml of 7.5% sodium carbonate solution is added to neutralize and alkalinize the reaction medium, followed by the formation of reduced polyphosphomolybdate, colored in blue. After 2 h of incubation in the dark room, the absorbance is read at 750 nm. The concentration of polyphenols is expressed using the calibration curve plotted at different concentrations of gallic acid. The analyzed samples (25 µl each) were dissolved in methanol and further dilution was effectuated to obtain readings within the standard curve made with gallic acid (R = 0.997). The extracts were oxidized by the Folin-Ciocalteu reagent (120 µl) and the neutralization was done with Na_2_CO_3_ (340 µl) after 5 min. Absorbance was read at 750 nm after 90 min in the dark at room temperature. The results were expressed in milligram of gallic acid/g dry matter of plant (mg GAE/g DW plant material).

### Total monomeric anthocyanin content

The differential pH method is the most widely used method for determining anthocyanin content [49, 51]. To obtain the extract, 3 g of *Prunus spinosa* pulp fine powder were weighed and homogenized in 10 ml of acidified methanol (85:15 v/v, MeOH:HCl) for 1 min at 20,000 RPM using a homogenizer (Ultra-Turrax Miccra D-9 KT Digitronic, Germany). Thus, the sample was centrifuged at 3500 rpm for 10 min. The extract was separated from the residue and re-extracted until the extraction solvent became colorless (total solvent volume was between 100 and 250 ml). The obtained extracts were combined in a total extract, and the moisture was removed using a rotary evaporator (R-124, Buchi, Switzerland) at 40 °C. In the dried samples 10 ml of methanol was added then centrifuged at 5000 rpm and filtered with a 0.45 µm filter (Millipore). Blackthorn extracts were dissolved in methanol then diluted with 0.025 mol/l potassium chloride (adjusted with HCl to pH 1.0) and 0.4 mol/l sodium acetate (pH 4.5). Each sample and standard (cyanidin-3-glucoside) was diluted with buffer solution pH = 1 and the absorbance was read at 520 nm and 700 nm, respectively, using a UV spectrophotometer (Jasco V-630, International Co Ltd, Japan). An aliquot of each sample was diluted to the same value with the buffer solution pH 5.0 and measured at 520 and 700 nm. The results were expressed as mg cyanidin-3-glucoside chloride per dry matter of plant (C3GE mg/g DW). Absorbance values were calculated according to the formula:

$${\text{A }} = \, \left( {{\text{A}}_{\lambda } 5 20 \, - {\text{ A}}_{\lambda } 700} \right)pH 1.0 \, - \, \left( {{\text{A}}_{\lambda } 5 20 \, - {\text{ A}}_{\lambda } 700} \right)pH 4. 5$$

Total anthocyanin content means were calculated as follows:

$${\text{TA}} = {\text{A}} \times {\text{MW}} \times {\text{DF}} \times {{1000} \mathord{\left/ {\vphantom {{1000} \varepsilon }} \right. \kern-0pt} \varepsilon } \times 1$$

## Results

### Biochemical analysis

The average values and standard deviation of blood serum parameters as AST, ALT, GLU, COL, TG and CRE obtained from biochemical analysis of blood are shown in Table 1.

**Table 1:** Average values and standard deviation for each biochemical parameter: AST, ALT, GLU, COL, TG and CRE

Parameter	Control	Group I	Group II	Group III
AST (U/l)	109.18 ± 0.92c	114.14 ± 1.44	108.42 ± 0.53c	119.88 ± 1.43a
ALT (U/l)	54.44 ± 0.60	62.42 ± 0.67a	56.85 ± 0.57	63.54 ± 0.66a
GLU (mg/dl)	181.96 ± 2.13c	191.76 ± 1.07	187.36 ± 1.07bc	200.24 ± 1.08a
COL (mg/dl)	67.95 ± 0.53	69.64 ± 0.24	68.28 ± 0.42	68.86 ± 0.58
TG (mg/dl)	51.68 ± 0.76c	54.7 ± 1.38	51.38 ± 0.94c	67.58 ± 1.33a
CRE (mg/dl)	0.41 ± 0.01c	0.49 ± 0.03ab	0.44 ± 0.01bc	0.53 ± 0.02a

Data represents the mean ± standard deviation (n = 5). Within columns, means followed by the same letter (a, b or c) are not significantly different between treatments according to the Tukey test (*p *= 0.05); While, within columns means followed by the different letter (a, b and c) are significantly different between treatments according to the Tuckey’s test (*p *= 0.05). ns: not significant; significant at the 0.05 probability level

The biochemical analysis of blood showed significant variations of GLU (mg/dl) depending on the tartrazine dose applied.

Significant values were obtained in the group III (200.24 ± 1.08) and the group I (191.76 ± 1.07) compared to the control group (181.96 ± 2.13). The values of total COL (mg/dl) did not show significant differences between groups. Regarding to TG (mg/dl), the significant values were observed in the group III (67.58 ± 1.33), which received the highest dose of food dye. TG values showed non-statistically significant differences among the control group, I and II (Table 1). According to Arefin et al. significantly increased levels of CRE and TG are reported in Swiss mice, after applying tartrazine dose 400 mg/kg b.w. by oral gavage [52].

In the present study AST (U/l) showed statistically significant means in the group I (114.14 ± 1.44) and III (119.88 ± 1.43) compared to the control group (109.18 ± 0.92) and the group II with fruit pulp powder (108.42 ± 0.53). The highest dose of tartrazine was able to significantly increase the level of ALT (U/l) for the group III (63.54 ± 0.66) and for the group I (62.42 ± 0.67) compared to the control group (54.44 ± 0.60), respectively. In contrary, the application of dried fruit powder mixed in drinking water in the (group II) determined a greater effectiveness reducing ALT (U/l) (56.85 ± 0.57) values, modifying them similarly to control group. Elevated levels of enzymes such as AST and ALT liberated into the bloodstream are directly correlated with damaged tissues of liver and heart [53]. Regarding CRE (mg/dl), the significantly increased values were registered for group III (0.53 ± 0.02) followed by the group I (0.49 ± 0.03), II (0.44 ± 0.01) compared to the control group (0.41 ± 0.01), respectively.

Similar results with the present study were found in the study realized by Himri et al. [54], who measured AST (U/l) (165.16 ± 17.82) and ALT (62 ± 2.84) in groups of rats that received a tartrazine dose of 10 mg/(kg b.w.) and was significantly higher compared to the control group (120 ± 5.64), (54.16 ± 5.33), respectively. According to results of Alaa Ali et al. [32], the addition of tartrazine in water could greater increase parameters as Urea, AST and ALT. Khayyat et al. reported significantly increased biomarkers means of ALT, AST, urea, and CRE after administering tartrazine to rats [28].

Moreover, El-Desoky et al. attributed the increase of concentrations of TG, COL, AST, ALT and CRE in blood serum due to the application of tartrazine in rats compared to the control group [55]. Furthermore, Al-Seeni et al. reported that applied doses of 10 mg/kg b.w. of tartrazine in rat diet, showed a significant increase in biochemical markers as: ALT (77.16 ± 1.68U/l), AST (79.16 ± 1.57U/l), ALP (275.83 ± 3.56U/l), and TG (224.67 ± 3.25 mg/dl) compared with the control group [56]. In addition, concurrent treatment of rats with *Nigella sativa* oil demonstrated a curative effect on biochemical and histological parameters.

### Hematological analysis

After the treatment period, the hematology investigations revealed significant changes in blood serum parameters for rats treated with tartrazine. The main results are presented in Table 2.

**Table 2:** Average values and standard deviation for hematological measurements of Wistar rats treated with tartrazine for 7 weeks

Parameter	Control	Group I	Group II	Group III
WBC 10^9^/l	4.8 ± 0.85ab	5.35 ± 1.38ab	3.85 ± 0.77b	5.87 ± 0.93a
LYM 10^9^/l	2.05 ± 0.44ab	2.79 ± 0.93a	1.55 ± 0.43b	3.06 ± 0.36a
MON 10^9^/l	0.45 ± 0.18a	0.15 ± 0.09b	0.26 ± 0.14ab	0.35 ± 0.21ab
NEU 10^9^/l	2.86 ± 0.53	2.61 ± 0.68	2.15 ± 0.37	2.47 ± 0.44
MO%	1.44 ± 0.24c	2.02 ± 0.70c	3.48 ± 0.25b	4.46 ± 0.26a
NE%	52.22 ± 4.54ab	46.98 ± 6.27bc	56.52 ± 3.65a	40.76 ± 1.40c
RBC 10^12^/l	7.86 ± 1.10	7.70 ± 0.59	7.82 ± 0.46	7.56 ± 0.69
HGB g/dl	14.4 ± 0.80a	12.46 ± 0.40a	13.68 ± 0.79b	12.56 ± 0.24b
HCT%	37.82 ± 5.10	36.97 ± 3.45	41.04 ± 2.21	37.07 ± 2.23
MCV fl	48.2 ± 1.30ab	47.2 ± 1.64b	50.6 ± 1.34a	50.2 ± 2.59ab
MCH pg	17.42 ± 0.88a	15.44 ± 0.51b	17.1 ± 0.64a	16.4 ± 1.27ab
MCHC g/dl	36.16 ± 1.28a	32.52 ± 1.29b	33.5 ± 0.41b	34.2 ± 1.64ab
RDWc%	18.52 ± 0.65a	18.72 ± 0.75a	16.74 ± 0.69b	17.14 ± 0.51b
PLT 10^9^/l	681 ± 69.56b	729.6 ± 32.25ab	866 ± 143.27a	502.6 ± 90.83c
PCT%	0.52 ± 0.09ab	0.41 ± 0.13b	0.65 ± 0.14a	0.33 ± 0.09b
LYM%	38.22 ± 3.05c	47.34 ± 4.62b	36.42 ± 1.54c	53.36 ± 3.08a
MPV fl	7.6 ± 0.57a	6.92 ± 0.13ab	7.32 ± 0.48ab	6.84 ± 0.29b
PDWc%	34.72 ± 0.97	33.2 ± 0.70	34.36 ± 1.40	33.36 ± 0.59

Data represents the mean ± standard deviation (n = 5). Within columns, means followed by the same letter (a, b or c) are not significantly different between treatment according to the Tuckey’s test (*p *= 0.05). While, within columns means followed by the different letter (a, b and c) are significantly different between treatments according to the Tuckey’s test (*p *= 0.05). ns: not significant;  significant at the 0.05 probability level

The percentages of LYM were very similar for the control group (38.22 ± 3.05) and the group II (36.42 ± 1.54), while the group I (47.34 ± 4.62) and III (53.36 ± 3.08) showed a significant increase in LYM% compared to the control group (Table 2). The significant differences were observed in MO% between the control group (1.44 ± 0.24) and the group III (4.46 ± 0.26) (Table 2), which received the highest intake (100 mg) of dye.

A significant decrease was observed in NE% count in the group III (40.76 ± 1.40) compared to the control (52.22 ± 4.54), whilst group II, which received blackthorn fruit powder, had more elevated percentage (56.52 ± 3.65) of NE compared to the control group. According to Himri et al., this food dye increased significantly the number of neutrophils in rat blood [54]. A significantly lower PLT (10^9^/l) count was found in the group III (502.6 ± 90.83) treated with 100 mg of tartrazine compared to the control group (681 ± 69.56). In the study of Himri et al., applying tartrazine at a dose of 7.5 mg/(kg b.w.) was able to significantly lower PLT (10^3^/µl) count (609.83 ± 45.58) compared to the control group (725.00 ± 11.56).

HGB (g/dl) in group II (13.68 ± 0.79), which received fruit extract, has approached values of the control group (14.4 ± 0.80) (Table 2). While, the groups I (12.46 ± 0.40) and III (12.56 ± 0.24) had a slight decrease of this measured parameter compared to the control group (Table 2). WBC (10^9^/l) increased significantly in the group treated with the highest dose of tartrazine compared to the control group (Table 2). There were no significant changes regarding LYM, MON, RBC, HCT, MCV, RDWc, PCT, MPV and PDW. An average amount of MCH (pg) found in the red blood cells of rats did not reveal significant changes in the group II (17.1 ± 0.64) compared to the control (17.42 ± 0.88). Moreover, MCH in the group I (15.44 ± 0.51) and group III (16.4 ± 1.27) showed a significant reduction compared to control and group II.

### Body weight and organ weight evaluations

In the present study, during the experiment no mortality cases were observed. The weights of the organs as liver, brain, spleen and kidneys are presented in Table 3.

**Table 3:** Average values and standard deviation of body weight and organ weight of experimental Wistar rats

Parameter	Control	Group I	Group II	Group III
Initial weight (g)	194.2 ± 5.0	195.6 ± 7.70	192.8 ± 4.32	193.8 ± 5.36
Final weight (g)	229.4 ± 9.11	231.1 ± 8.97	229.8 ± 3.77	239.4 ± 7.60
Liver (g)	7.87 ± 0.58	8.4 ± 0.76	8.13 ± 0.51	8.83 ± 0.30
Right kidney (g)	0.75 ± 0.45c	0.91 ± 0.08b	0.81 ± 0.07bc	1.108 ± 0.69a
Left kidney (g)	0.76 ± 0.06c	0.92 ± 0.07b	0.82 ± 0.06bc	1.106 ± 0.69a
Spline (g)	0.62 ± 0.04	0.64 ± 0.17	0.60 ± 0.08	0.57 ± 0.03
Brain (g)	1.60 ± 0.14ab	1.44 ± 0.04b	1.52 ± 0.06b	1.76 ± 0.15a

Data represents the mean ± standard deviation (n = 5). Within columns means followed by the same letter are (a, b or c) are not significantly different between treatments according to the Tuckey’s test (*p *= 0.05). While, within columns means followed by the different letter (a, b and c) are significantly different between treatments according to the Tuckey’s test (*p *= 0.05)

Body weight and relative weight of the liver, kidneys, brain and spline of rats for the group III, which received a high dose of tartrazine were slightly increased compared to other groups. After 7 weeks of exposure of animals to tartrazine there were no significant weight differences observed between the final weight of the groups. Kidneys, brain and liver weights showed differences among the groups (Table 3).

### Histological evaluation

**Fig. 2:**
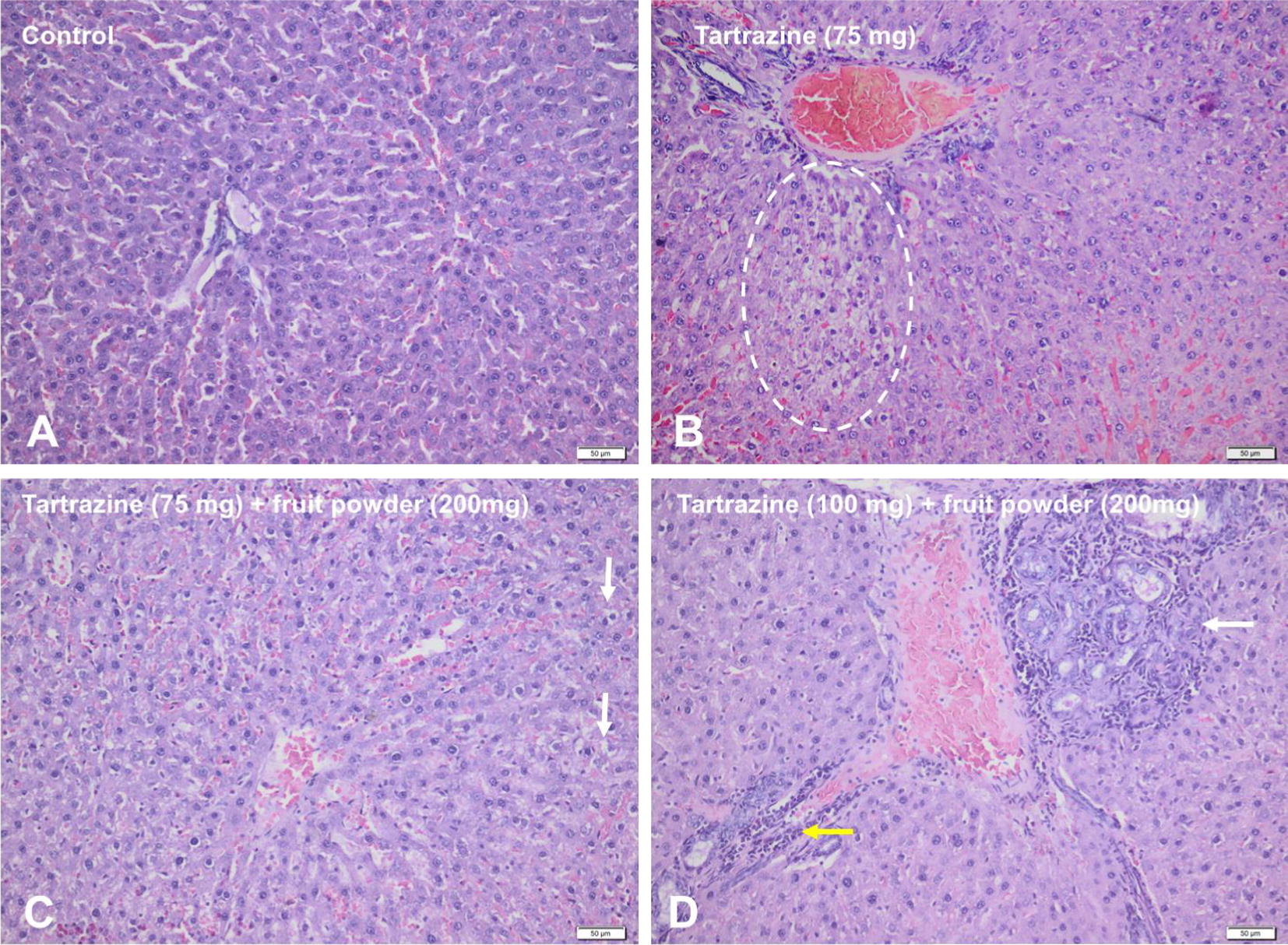
Microscopical findings of the liver from experimental albino Wistar rats. **A** Normal features in control group. **B** Hepatic congestion and hepatocellular vacuolar degeneration and necrosis of the periportal area (delimited zone), group I. **C** Hepatic congestion and mild vacuolar degeneration of the periportal hepatocytes (white arrows), group II. **D** Bile duct hyperplasia (white arrow) and mixed inflammatory infiltration of the portal tract, group III (yellow arrow). Hematoxylin–Eosin (H&E) stain

In the control group, no important structural changes were noted in the liver (Fig. 2A), spleen, kidneys and brain.

In the group I, treated with 75 mg of tartrazine diluted in 250 ml of water, the microscopical examination showed severe hepatic congestion, mild Kupffer cell proliferation, vacuolar degeneration of the hepatocytes and individual cell necrosis in the periportal area, bile duct hyperplasia, mild fibrosis and periductal mixed inflammatory cell infiltration (Fig. 2B). The histological changes of kidneys were mild and moderate and consisted of glomerular congestion, interstitial congestion and edema, and mild vacuolar degeneration of renal proximal convoluted tubules (personal communications Balta et al.). No significant changes were identified in the spleen and brain. In group II, treated with 75 mg of tartrazine and 200 mg of fruit powder, the hepatic changes were significantly decreased and included hepatic congestion, mild Kupffer cell hyperplasia and mild periportal vacuolar degeneration (Fig. 2C). Kidney tissues of the group I, showed mild renal congestion and hydropic degeneration of renal tubular epithelium (personal communications Balta et al.). No significant changes were identified in the other examined organs.

In group III, treated with 100 mg of tartrazine and 200 mg of fruit powder, the histological changes of the liver and kidneys were significantly higher compared to the other groups. The hepatic parenchyma showed severe bile duct hyperplasia, portal inflammation and mild fibrosis (Fig. 2D). The kidneys showed glomerular congestion, mild interstitial edema and mixed inflammatory infiltration and vacuolar degeneration of proximal convoluted tubules (personal communications Balta et al.). In the spleen of the rats in group III, numerous lymphoid hyperplasia, hemosiderin-laden macrophages and extramedullary hematopoiesis were identified (personal communication Balta et al.). Microscopical examination of the brain tissue revealed congestion, spongy status of the cerebral gray matter and mild gliosis (personal communications Balta et al.).

### Total polyphenolic and anthocyanin content

**Fig. 3:**
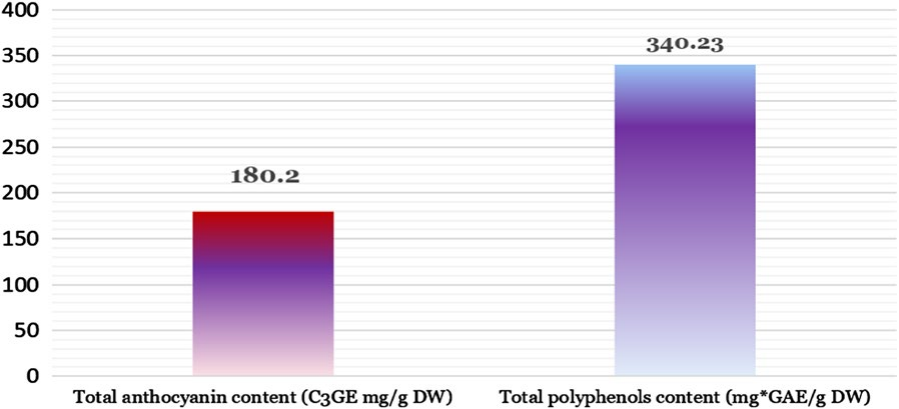
Total anthocyanin and polyphenols content from *Prunus spinosa* fine pulp powder

Total anthocyanins and polyphenols content from blackthorn fruits are presented in (Fig. 3). The total polyphenols content was revealed using the Folin–Ciocalteu method [49, 50] and total monomeric anthocyanin content was determined by the differential pH method. The total polyphenolic and anthocyanin content in analyzed Blackthorn extract presented values of 340.23 (mg GAE/g DW) and 180.2 (C3GE mg/g DW), respectively.

## Discussion

The most significant changes of the liver and kidneys specimens after 7 weeks of tartrazine exposure were observed in group III. Our deduction is that this dye can act through stasis. The application of the highest dose (100 mg diluted in 250 ml of water) was able to cause hepatic congestion, bile duct hyperplasia and mixed infiltration into the portal spaces in the liver of group III. In a study, tartrazine demonstrated toxic effects on male albino rats, presenting diffuse vacuolar degeneration in the hepatic parenchyma, hepatic congestion and hyperplasia in the bile duct epithelium, and in the hepatocytes of some individuals, necrobiosis was detected [32].

Histopathological results from the present study are in concordance with those reported by Al-Seeni et al. that observed atrophic tubular degeneration with lining and congested glomeruli in kidney tissue and broad infiltration of the lymphocytes in the liver of the tartrazine treated rats compared to the control group [56]. A study conducted by Ghonimi et al. aimed to test the sub-chronic toxicity of tartrazine and the protective effects of both royal jelly and cod liver oil in some parenchymal organs of Wistar rats [57].

After application of tartrazine theraphy for 30 days at dose of 500 mg/kg by gavage, histological investigations showed modifications in the liver, brain, stomach, kidneys and testes. The liver was damaged, and necrosis of hepatic tissue, severe steatosis, diffuse degeneration, fibrous tissue proliferation with anti-inflammatory cells in the portal area and moderate disorganization of hepatic cords was showed. In the group II, testis examination showed proliferation and hyperplasia of the interstitial Leydig cells with vacuolations. Furthermore, vacuolization in the brain tissues, especially in white matter and degenerative changes in the stomach mucosa was observed. In the kidneys, hyaline degeneration was detected in renal tubules and vacuolations of glomeruli. In addition, the protective effect of royal jelly and cod liver oil resulted in a statistically non-significant reduction of tartrazine toxicity. In a study realized by El-Desoky et al. showed a dilation of blood sinusoids and central vein with hemorrhage and necrosis in the liver of rats exposed to this dye [55]. Interestingly, in a similar study of Hoseinpouran et al. onion (*Allium cepa*) added in the rat diet reduces the toxic effect of tartrazine on the enzymatic activity of antioxidants and biochemical serum parameters [21].

Applying daily doses of 0–7.5 mg/kg/day might produce neurotoxicity and a deficiency in the learning and memory process in rodents, due generation of reactive oxygen species and lipid peroxidation metabolites [58]. According to Aboel-Zahab et al. results, rats, which received a diet supplemented with a mixture of food dyes (such as sunset yellow, tartrazine, carmoisine, and brilliant blue) in different concentrations showed a significant increase in total serum lipids, COL, total protein and globulin serum transaminases [59]. Additionally, hematological analysis showed selective neutropenia and lymphocytosis, eosinophilia, and a significant decreased in hemoglobin concentration and RBC count. Histopathological investigations revealed congestions of blood vessels, areas of hemorrhage in both, renal and liver sections and brown pigment deposition in liver Kupffer cells. As reported by Sasaki et al. using a comet assay, tartrazine that was applied at a dose of 7.5 mg/kg could induce DNA damage in the colon of mice [22]. Nabila Mehedi et al. concluded that tartrazine applied in drinking water at the different low doses induced depression of body weight and adverse effects on the organs of Swiss mice [31]. Moreover, lesions in the brain, liver, and kidneys in animals treated with tartrazine were observed. The number of red blood cells, hemoglobin and hematocrit was increased, while the number of white blood cells was decreased in males of all groups. The excessive consumption of food dyes may cause problems for children’s health.

In another study carried out by Mehedi et al. on Swiss albino mice it was observed that the excessive consumption of tartrazine could produce adverse effects on fertility, lowering sperm counts and increasing sperm abnormalities [60]. In this study, tartrazine was added at different doses (0, 0.1, 1, and 2.5%) in drinking water, for adult male mice during 13 weeks. Himri et al. reported that sulfanilic acid at dose of 3.75 mg/kg b.w. and tartrazine at dose of 10 mg/kg b.w. was able to trigger a morphologic change in red blood cells of rats, respectively modifying the discoidal form cell to an echinocytic shape [54]. Moreover, the liver weights, AST, CHL, CRE, GLU, TG and total protein in serum of the Wistar rats were increased significantly. Histopathological investigations showed changes that have occurred in kidneys as tubular dilatation, tubular degeneration, dilatation of the glomerular capillaries, intercapillary sclerosis, and atrophy of glomerulus. The author concluded that tartrazine does not only produced changes in the liver or kidney parameters, but also the impact of this dye becomes more dangerous when higher doses are applied, because it can induce oxidative stress by formation of free radicals.

In the study realized by Golli et al. [61], the food dye was administered orally at a dose of 300 mg/kg/b.w. to rats. However, Golli et al. [61] observed an increase in thrombocytes counts in rats and a dose of 300 mg/kg/b.w. of tartrazine was able to elevate the activity of hepatocellular enzymes facilitating changes in kidney biomarkers. On the other hand, peripheral lymphocytes and spleen T CD8-lymphocytes were reduced, suggesting the toxic potential of tartrazine on immune response, hepatic and renal functions. Furthermore, critical oxidative modifications in all analyzed organs were revealed due to the promotion of lipid peroxidation and the changes in endogenous antioxidant-defense enzymes. Therefore, the author concluded that sub-chronic exposure of tartrazine could produce deficiencies in blood parameters, immune-toxicity leading to renal and hepatic impairment by modifying the total equilibrium between oxidants and antioxidants [61].

## Conclusions

Altogether, our results showed that tartrazine was able to generate histopathological changes, which caused certain tissue lesions of the liver, kidney, and spleen. The administration of this dye in the diet of rats can induce significant changes in all biochemical and hematological parameters of the blood. Notably, we elucidated the promising protective role of Blackthorn (*Prunus spinosa*) fine powder on hematological and biochemical parameters of blood, added in Wistar albino rats diet. On the other hand, fruit powder demonstrated non-significant protective effects for analyzed organs.

## Data Availability

The data supporting the conclusions have been presented in the research paper.

## Abbreviations

B.w.: body weight
EDTA: ethylenediaminetetraacetic acid
AST: aspartate aminotransferase
ALT: alanine aminotransferase
GLU: glucose
COL: cholesterol
TG: triglyceride
CRE: creatinine
LYM%: percentage of lymphocytes
RBC: red blood cells
HGB: hemoglobin concentration
HCT: hematocrit
MCV: corpuscular volume
MCHC: corpuscular hemoglobin concentration
LYM: small/medium lymphocytes
MCH: corpuscular hemoglobin
WBC: white blood cells
MPV: platelet volume
MO%: relative percentage content of monocytes
NEU: contents of neutrophils
NE%: relative (%) content of neutrophils
RDW: red cell distribution width
PCT: represents plateletcrit
PDW: platelet distribution width
DW: dry weigh
C3GE: cyanidin-3-glucoside chloride
GAE: gallic acid
